# Leveraging *in vivo* animal models of tendon loading to inform tissue engineering approaches

**DOI:** 10.3389/fbioe.2024.1449372

**Published:** 2024-10-07

**Authors:** Samantha Muscat, Anne E. C. Nichols

**Affiliations:** ^1^ Department of Pathology and Laboratory Medicine, University of Rochester School of Medicine and Dentistry, Rochester, NY, United States; ^2^ Department of Orthopedics and Physical Performance, Center for Musculoskeletal Research, University of Rochester Medical Center, Rochester, NY, United States

**Keywords:** tendon, loading, tissue engineering, adaptation, disuse, underloading, overuse

## Abstract

Tendon injuries disrupt successful transmission of force between muscle and bone, resulting in reduced mobility, increased pain, and significantly reduced quality of life for affected patients. There are currently no targeted treatments to improve tendon healing beyond conservative methods such as rest and physical therapy. Tissue engineering approaches hold great promise for designing instructive biomaterials that could improve tendon healing or for generating replacement graft tissue. More recently, engineered microphysiological systems to model tendon injuries have been used to identify therapeutic targets. Despite these advances, current tissue engineering efforts that aim to regenerate, replace, or model injured tendons have largely failed due in large part to a lack of understanding of how the mechanical environment of the tendon influences tissue homeostasis and how altered mechanical loading can promote or prevent disease progression. This review article draws inspiration from what is known about tendon loading from *in vivo* animal models and identifies key metrics that can be used to benchmark success in tissue engineering applications. Finally, we highlight important challenges and opportunities for the field of tendon tissue engineering that should be taken into consideration in designing engineered platforms to understand or improve tendon healing.

## Introduction

Tendons are dense, connective tissues that connect muscle to bone to facilitate movement within the musculoskeletal system. Though frequently injured, either through acute injury resulting from trauma or through degenerative processes (i.e., tendinopathies), current treatments are largely conservative and do not adequately address the underlying cellular dysfunction that is at the heart of the failed healing response (Irby et al., 2020). Tissue engineering offers an attractive alternative to traditional treatment modalities that could help recover, improve, and maintain tendon function (Lim et al., 2019; Ruiz-Alonso et al., 2021; Ning et al., 2023). The overall goal of tissue engineering is to regenerate, replace, or model pathological tendon tissues. The typical tissue engineering approach has been to combine various types of cells, biomaterials, bioactive molecules, and mechanical loading to try and generate tendon tissue; however, current efforts have largely failed to successfully recreate the complex tendon architecture due to an incomplete understanding of the required mechanical strain, appropriate cellular behavior and composition, spatial complexity, and tendon-specific considerations needed to model healthy or diseased tendons. More recently, tissue engineering constructs have also been used to model tissue dysfunction in the form of microphysiological systems (MPS) or “organ-on-a chip” models. These models strive to be physiologically relevant, often combining multiple compartments seeded with different interacting cell types to model either healthy tissue or pathological processes (Wang et al., 2020; Awad et al., 2023). However, largely absent from current tendon MPS is the role of mechanical strain in promoting or preventing tendon dysfunction. Though there is still much to be discovered and clarified regarding the specific cell populations, mechanisms, and structure/function relationships guiding tendon function, *in vivo* animal models can offer important insight into how altered mechanical loading affects tendon cell behavior, and how this translates to a tissue-level response. The goal of this review is to summarize the findings of *in vivo* animal models of tendon under- and over-loading and to identify key structural, histological, mechanical, and material outcomes that can be used to guide and inform the design of engineered tendon constructs and MPS. Importantly, we also highlight how a lack of understanding of key biological features of tendons impedes tissue engineering progress and identify crucial areas for future study that will enhance our ability to design accurate tendon tissue mimetics.

### Proper tendon function is dependent on maintenance of a complex extracellular matrix

Critical to tendon function is the ability to withstand and recover from extreme loads, a quality that is imparted to the tissue due to the highly aligned extracellular matrix (ECM) which makes up the body of the tendon. Collagen type I is the primary constituent of the tendon ECM and comprises ∼60%–85% of the tendon dry weight (Screen et al., 2015). In addition to the collagen matrix, proteoglycans and glycosaminoglycans (GAGs) provide structural support and, by increasing water content, provide resistance to compressive force (Thorpe et al., 2013). Beyond the collagen matrix, tendons of larger mammals including humans possess a hierarchical structure wherein individual fascicles (bundles of collagen fibrils) are surrounded by other types of ECM, including the interfascicular matrix (IFM) (Patel et al., 2021) and the poorly defined endotenon (Kannus, 2000), which is surrounded by a thin, loose connective tissue called the epitenon (Nichols et al., 2023). The endotenon and epitenon are contiguous structures that house the vasculature and nerves that supply the tissue with nutrients and innervation. This complex matrix and structure allows tendons to transmit forces, dissipate energy, and prevent mechanical failure without compromising structural integrity (Franchi et al., 2007). Tendons, both healthy and injured, are frequently assessed by measuring the biomechanical properties of the tissue, which gives information on both the structural properties (e.g., stiffness, maximum load) and material quality (e.g., peak stress, elastic modulus) of the tendon. While outside the scope of this review, we point readers towards several excellent papers on the mechanical properties of tendons and means of assessment (Lomas et al., 2015; Lake et al., 2023). Cross-sectional area (CSA) is also frequently used as a metric of tendon health; however, whether in CSA are reflective of natural adaptive processes or the development of tendinopathy is unclear and likely both context- and time-dependent. Importantly, the composition of the ECM, and therefore the mechanical properties, of the tendon is dependent on the particular tendon type. For example, human Achilles tendons have increased sulphated GAG and water content compared to the anterior tibialis tendon (Birch, 2007) and lower water content is associated with a stiffer tendon (Birch, 2007), which suggests that the properties of any tendon must be considered in the proper anatomical context.

In healthy tendons, the primary resident tendon cell is a specialized type of fibroblast called the tenocyte, or tendon fibroblast. Tenocytes are interspersed sparsely throughout the tendon ECM, and appear as thin, spindle-shaped, cells oriented along the axis of tension in linear arrays. Tenocytes are thought to be the main cellular players that maintain tendon homeostasis. In response to appropriate (e.g., physiological) mechanical load, tenocytes facilitate matrix turnover and repair by upregulating the production of growth factors (e.g., transforming growth factor-β, fibroblast growth factor-2, etc.), collagen synthesis enzymes, and matrix metalloproteinases (MMPs) which ultimately preserves the integrity of the tendon ECM (Birch, 2007; Franchi et al., 2007; Subramanian et al., 2018). Precisely how tenocytes sense and respond to mechanical stimulation, or how this cellular behavior results in tissue-level changes to the tendon, is unknown. Similar to the ECM composition, the cellular density (Birch, 2007) and specific tenocyte populations present vary between tendons (Kendal et al., 2020), reinforcing the idea that different tendons must be considered as distinct entities. In addition to tenocytes, recent studies have reported the presence of innate (e.g., macrophages) and adaptive (e.g., T cells) immune cells (Kendal et al., 2020; Garcia-Melchor et al., 2021; Muscat et al., 2022; Bautista et al., 2023) within healthy tendons, though their role in tendon homeostasis, or how these populations might vary tendon to tendon is unclear. Interestingly, recent work has suggested that resident immune cells have mechanosensing capabilities in other tissues, and that changes in the local mechanical environment can activate immune cells to an inflammatory phenotype (Meli et al., 2020; Zhang et al., 2020). Whether resident immune cells play a similar role in the tendon response to loading is unknown but warrants further investigation.

### Improper mechanical stimulation is a primary driver of tendinopathy

Tendinopathy is a broad term that describes a slow, degenerative process that compromises the integrity of the organized tendon ECM, leading to reduced function, limited mobility, and pain (Steinmann et al., 2020; Millar et al., 2021; Chatterjee et al., 2022). Tendinopathy is complex and multifactorial as age, sex, activity level, and obesity are all known risk factors that can increase the risk of developing tendinopathies (Millar et al., 2021); however, improper mechanical loading is believed to be the overarching primary driver of tendinopathy. Tendinopathies due to overload or overuse are thought to be a failed healing response, where supraphysiological (overload) or repetitive injurious loads (overuse) cause damage to the ECM that tenocytes are unable to repair. This leads to further damage and degeneration due to the inability of the compromised tendon to withstand the forces it experiences (Millar et al., 2021). Conversely, tendon underloading can prevent proper stimulation of matrix turnover by tenocytes which weakens the organized matrix of the tendon, leading to a similar degenerative cycle as overload injuries (Herod and Veres, 2018). As such, tendinopathic tendons frequently exhibit altered mechanical and material properties compared to healthy tendons (Arya and Kulig, 2010).

In addition to structural changes, tendinopathic tendons are generally characterized histologically by disorganization of the collagen matrix, increased staining for proteoglycans/GAGs, increased cellularity, and in some cases, increases in the presence of sensory nerve fibers and microvasculature within the body of the tendon (Fredberg and Stengaard-Pedersen, 2008), though as we detail below, the specifics of these tendinopathic changes are both tendon- and context-dependent. Tenocytes present in tendinopathic tendons can exhibit changes in cellular morphology that include loss of the linear cellular alignment and cell rounding (Fredberg and Stengaard-Pedersen, 2008). Tendinopathic tenocytes also exhibit greater transcriptional diversity compared to uninjured tenocytes (Kendal et al., 2020; Millar et al., 2021; Mimpen et al., 2024a), and increased expression of cell activation markers including alpha smooth muscle actin (a myofibroblast marker) (Kendal et al., 2020). Historically, the role of inflammation in human tendinopathy has been debated as late-stage tendinopathic tendons (when most samples are collected) frequently do not exhibit the presence of immune cells (Jomaa et al., 2020). However, other work has shown a role for inflammation in tendon degeneration (Millar et al., 2017). Tendinopathic human Achilles tendon biopsies exhibited an inflammatory signature marked by increased numbers of CD14+ and CD68+ myeloid cells and upregulation of pro-inflammatory NF-kβ and interferon pathways compared to healthy tendons (Dakin et al., 2018), which suggests that chronic inflammation may be involved in tendinopathic progression. This is supported by a rat model of collagenase-induced Achilles tendinopathy, which also showed evidence of non-resolving inflammation and accumulation of CD163+ macrophages (Marsolais et al., 2001). In addition to macrophages, T cells have been observed in tendinopathic samples from human rotator cuff, and coculture experiments demonstrated that tenocytes and T cells can stimulate each other to a pro-inflammatory phenotype (Garcia-Melchor et al., 2021). Whether these immune cells arise from resident cells or are recruited from the blood is unknown. Combined, these data suggest that both immune cells and tenocytes can promote an inflammatory environment that may lead to the development of tendinopathy, however the specific mechanisms by which this occurs remain to be determined.

**FIGURE 1:**
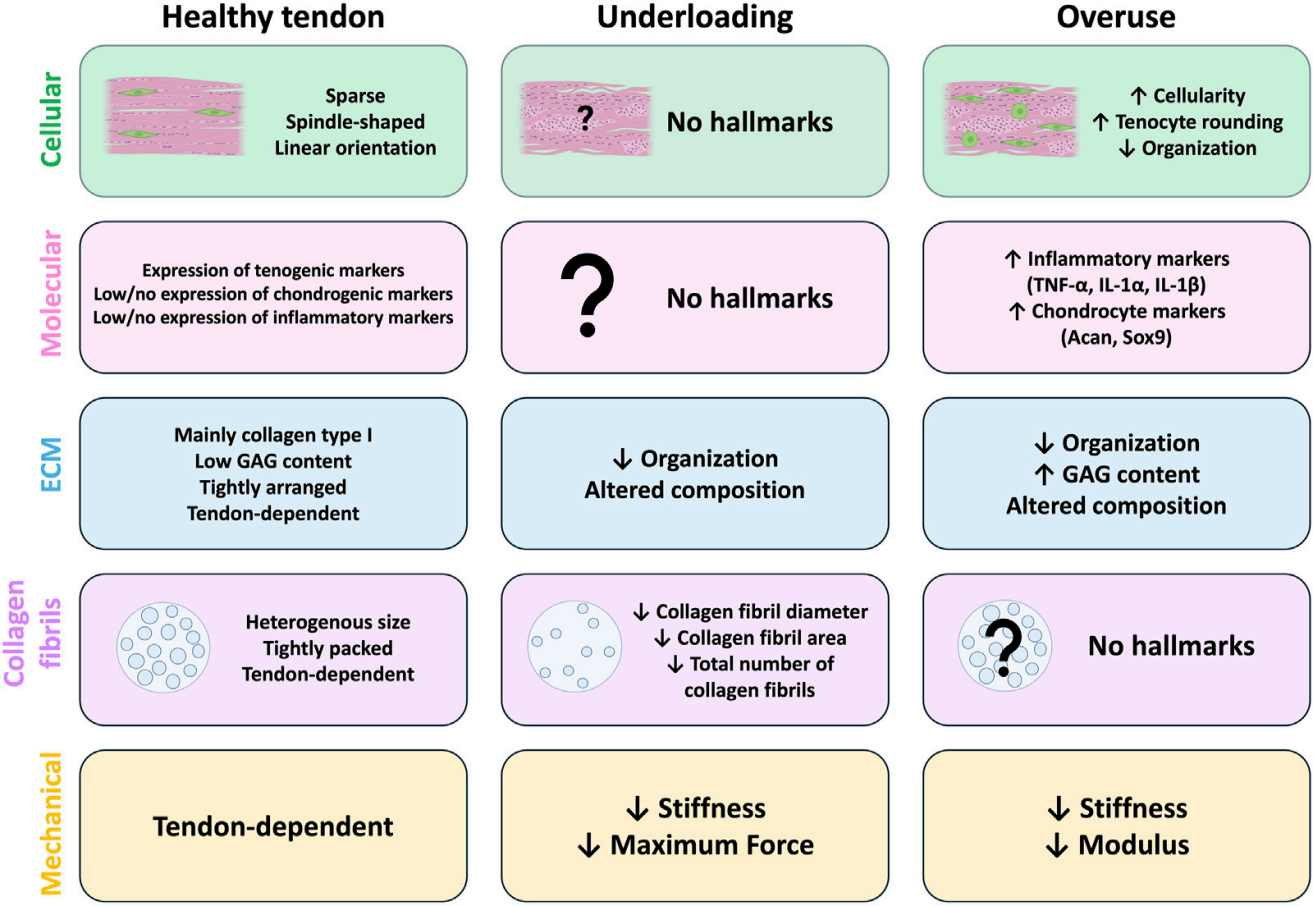
Hallmarks of tendon underloading and overuse compared to healthy tendons. Common cellular (green), molecular (pink), extracellular matrix (ECM; blue), collagen fibril (purple), and mechanical (yellow) hallmarks identified in animal models of healthy tendons (left) and those with underload (middle) or overuse-induced (right) tendon degeneration. In healthy tendons (left), tenocytes are sparse and arranged in linear arrays along the axis of tension. Healthy tendons typically have high expression of tenogenic markers and low/no expression of chondrogenic or inflammatory markers. The exact composition of the ECM is tendon-dependent, but the ECM of healthy tendons is highly organized, composed primarily of collagen type I with low glycosaminoglycan (GAG) content. Collagen fibrils are heterogenous in size and tightly packed, however the size composition is tendon-dependent. Mechanical and material properties of healthy tendons are also tendon-dependent. No studies report any cellular or molecular hallmarks of tendon underloading (middle). Underloading generally leads to altered ECM composition and decreased organization compared to healthy tendons. The overall number of collagen fibrils is reduced and the individual fibrils themselves are smaller, and underloaded tendons exhibit reduced stiffness and max force compared to healthy tendons. (Right) Models of tendon overuse demonstrate cellular hallmarks such as increased cellularity, tenocyte rounding, and decreased cellular organization. Molecular hallmarks include increased expression of inflammatory (TNF-α, IL-1 α, IL-1 β) and chondrocyte (Acan, Sox9) markers compared to healthy tendons. The ECM composition of overused is altered, with increased GAG content and decreased organization. Collagen fibril density and size alterations from overloading models are not reported. Overuse tendons exhibit decreased stiffness and elastic modulus compared to healthy tendons.

It is clear that proper mechanical stimulation is required for tendon health, as too much or too little can lead to tissue degeneration. Therefore, in order for tissue engineering to create new tendon tissue (proper loading) or to model tendinopathy (underloading or overuse), it is crucial to identify multiscale features that can be used to assess failure or success of engineered constructs. Below, we outline what is known about both underloading and overuse injuries in human tendons, describe animal models that aim to replicate the human conditions, and leverage these findings to establish cellular, molecular, and structural hallmarks that can be used to guide future tendon tissue engineering efforts (Figure 1).

### Tendon underloading in humans

Tendon underload is defined as the disruption of mechanical stimuli that causes a loss of load transmission to resident tendon cell populations. In humans, tendon underuse can occur during bedrest (Kubo et al., 2004; Reeves et al., 2005) or can be modeled by unilateral limb suspension via casting (Christensen et al., 2008; Couppe et al., 2012) or suspension with crutches in healthy individuals (de Boer et al., 2007a; de Boer et al., 2007b). In all cases, underloading appears to result in impaired mechanical and material properties without large structural changes. Achilles tendons of bed-rested volunteers had reductions in stiffness (58%) and elastic modulus (57%) after 90 days compared to pre-bed rest measurements with no changes in CSA (Reeves et al., 2005). Most human underuse studies see no changes in CSA but this could potentially be attributed to short periods of disuse (<90 days) (Kubo et al., 2004; Reeves et al., 2005; de Boer et al., 2007a; de Boer et al., 2007b; Christensen et al., 2008). With chronic disuse (at least 1.5 years), as seen in paralyzed patients from a previous spinal cord injury, the patellar tendon exhibited significant reductions in CSA (17%), stiffness (77%), and elastic modulus (59%) compared to able-bodied controls (Maganaris et al., 2006). Taken together, these human underuse studies suggest that impairments in mechanical and material properties occur early within the first few months of immobilization. Longer lengths of underloading are necessary to induce large structural changes in the tendon (i.e., changes in CSA).

**FIGURE 2:**
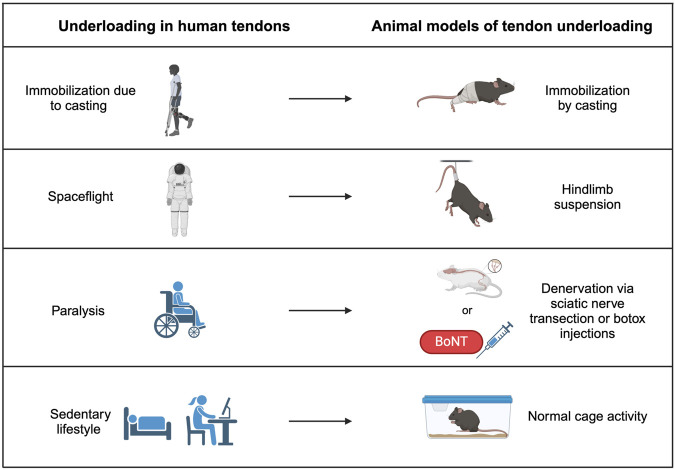
Tendon underloading in humans and corresponding animal models. Immobilization from a cast due to another injury causes underloading in tendon and can be modeled by casting the hindlimbs of animals. Loss of gravity observed in spaceflight can cause underloading in tendons of astronauts and is modeled by hindlimb suspension in animal models. Paralysis from a previous injury causes underloading in human tendons and can be replicated by either transecting the sciatic nerve or injecting Botulism toxin (Botox), which both prevent muscle contraction and therefore reduce load on the tendon. Sedentary lifestyles promote underloading in human tendons and may be modeled in mice by normal cage activity.

While decreases in stiffness and elastic modulus with tendon disuse are consistent in human models of underloading, animal models are sparse and inconsistent. In this next section, we describe efforts to establish animal models of tendon disuse that model human equivalents (Figure 2). Specifics regarding the models and outcomes of all studies outlined below are provided in Table 1.

**TABLE 1:** Models of tendon underloading and their reported outcomes used to identify common hallmarks of tendon underloading. Gray boxes indicate outcome was not reported.

Model	Animal	Tendon	Cellular density	Cellular morphology	Extracellular matrix organization	Collagen fibril size	Collagen fibril number	Tendon size	Inflammation markers	Chondrocyte markers	Mechanical properties	Material properties	Vasculature	References
Cast	Rabbit	Achilles			No change			No differences in CSA			Decreased stiffness and peak load			Matsumoto et al. (2003)
	Rabbit	Patellar												Harwood and Amiel (1992)
	Rabbit	Tibilas Anterior						No differences in CSA			Decreased stiffness and ultimate strength	Decreased maximum stress		Loitz et al. (1989)
Chemical denervation (Botox)	Guinea Fowl Keet	Achilles						No differences in CSA			No differences in stiffness	No differences in modulus		Katugam et al. (2020)
	Mouse	Patellar			Disorganized			Decreased tendon width						Chen et al. (2021)
	Mouse	Supraspinatus			Disorganized			Decreased CSA			Decreased maximum force, strength, and stiffness	Decreased modulus		Schwartz et al. (2013)
	Rat	Achilles						No differences in transverse area			No differences in stiffness or peak force	Increased modulus and decreased creep		Eliasson et al. (2007)
	Rat	Achilles			No change						Increased stiffness	Decreased creep and hysteresis Increased stress relaxation		Khayyeri et al. (2017)
Denervation	Rat	Achilles									Increased stiffness			Vilarta and Vidal (1989)
	Rat	Achilles	Increased	Rounded	Disorganized									El-Habta et al. (2018)
	Rat	Tibilas anterior						Increased tendon mass			Increased stiffness	Increased modulus		Arruda et al. (2006)
Hindlimb suspension	Rat	Achilles									Decreased stiffness	Decreased maximum stress and modulus		Almeida- Silveira et al. (2000)
	Rat	Achilles				Decreased	Decreased	No significant differences in CSA						Nakagawa et al. (1989)
	Rat	Patellar tendon			Disorganized									Valias et al. (1988)
Spaceflight	Rat	Achilles enthesis				Decreased	Decreased	Decreased						Deymier et al. (2019)
	Rat	Supraspinatus enthesis									No differences in stiffness or maximum force			Deymier et al. (2019)

### *In vivo* animal models of tendon underloading

#### Immobilization

To replicate tendon underloading due to casting in humans, animal models of limb casting are frequently used. In a rabbit model in which a fiberglass cast was used to prevent mechanical loading of the Achilles tendon, 4 weeks of immobilization resulted in significantly reduced stiffness (64%) and peak load (14%) with no changes to the CSA or collagen alignment (Matsumoto et al., 2003). Similarly, studies of patellar (Harwood and Amiel, 1992) and tibialis anterior tendon (Loitz et al., 1989) immobilization by casting report decreases in stiffness and ultimate strength, while changes in CSA or collagen organization were not examined.

#### Hindlimb suspension

Hindlimb suspension is a popular model to reduce tendon loading in animal models and is frequently used to mimic physiological responses due to lack of mechanical loading seen during spaceflight (Morey, 1979; Morey-Holton and Globus, 1998). While 20 days of hindlimb suspension is sufficient to enact structural changes in bone (Morey, 1979), hindlimb suspension models in tendon require longer durations (three to 5 weeks) (Vailas et al., 1988; Nakagawa et al., 1989; Almeida-Silveira et al., 2000). In the Achilles tendon, collagen fiber diameter decreased by 23.1% after 5 weeks of hindlimb suspension in Wistar rats compared to controls (Nakagawa et al., 1989). No changes in Achilles CSA were detected after 3 weeks of hindlimb suspensions in rats; however, both stiffness (41.5%) and maximum stress (37.4%) were significantly decreased compared to control tendons (Almeida-Silveira et al., 2000).

#### Denervation

To model tendon paralysis due to nerve injury in human patients, denervation via transection of the sciatic nerve in animals, which leads to limb tendon unloading, is a common model. Denervation of the sciatic nerve resulted in a 291% increase in the stiffness of rat tibias anterior tendons (Arruda et al., 2006). Disorganization of newly deposited collagen, hypercellularity, and rounded cell morphology suggest unilateral tendon unloading via sciatic nerve transection was sufficient to induce pathologic changes in rat Achilles tendons after 2 weeks (El-Habta et al., 2018).

Chemical denervation using Botulism toxin type A (Botox) injections is minimally invasive and has become a popular choice to immobilize lower extremities in rodents. Botox is a non-lethal neuromuscular blocking agent that targets motor nerve terminals in muscle with high specificity by blocking acetylcholine release, preventing action potentials and therefore muscle contraction (Brin, 1997). Studies using Botox as a model of disuse have established that muscle contraction is necessary to maintain bone mass and strength (Chappard et al., 2001; Warner et al., 2006; Grimston et al., 2007; Bach-Gansmo et al., 2016; Brent et al., 2020); however, unloading by Botox injections has yielded various results in both anatomically distinct tendons and different animals models, suggesting both a species and tendon-specific response to Botox unloading. In the rat Achilles tendon, Botox unloading lead to a 45% increase in elastic modulus and a 19% decrease in hysteresis (Eliasson et al., 2007; Khayyeri et al., 2017) with no change in CSA (Khayyeri et al., 2017) or tendon length (Eliasson et al., 2007). Other studies have reported both increased (Khayyeri et al., 2017) and no change (Eliasson et al., 2007) in rat Achilles tendon stiffness with Botox unloading. In contrast, Botox unloading of the mouse patellar tendon resulted in significantly reduced tendon width (Chen et al., 2021). The enthesis of the supraspinatus tendon demonstrated a 25% decrease in CSA and a 80% decrease in yield stress following Botox unloading (Schwartz et al., 2013). Botox unloading in avian models such as guinea fowl keets demonstrated no changes in CSA, stiffness, elastic modulus and hysteresis (Katugam et al., 2020).

### Hallmarks of tendon underloading

Despite the relatively limited number of studies, it is clear that inadequate mechanical stimulation leads to altered tendon biomechanical properties; however, the magnitude and specific alterations appear to be tendon-dependent. Most short-term studies reported no changes in CSA, suggesting that long-term or chronic disuse is necessary for large-scale structural changes. Instead, reductions in collagen fiber area and diameter (Nakagawa et al., 1989) could explain mechanical and material deficits (Loitz et al., 1989; Almeida-Silveira et al., 2000; Matsumoto et al., 2003; Schwartz et al., 2013) seen with *in vivo* animal models of unloading. Absent from the unloading animal studies are models that replicate bedrest, or a sedentary lifestyle; however, investigators should consider that normal cage activity as seen in lab mice may be a model of unloading, given that wild mice typically run long distances (Meijer and Robbers, 2014) and when given voluntary access to running wheels, laboratory mice will run up to 20 km per day (Manzanares et al., 2018). Additionally, there are clear differences in how different tendons respond to various types of unloading. As such, it is not possible to identify a “one-size-fits-all” strain rate that will result in tendon unloading. Investigations aiming to create healthy tendon mimetics should therefore focus on identifying homeostatic levels of mechanical strain within a given tissue engineering or MPS system that will result in a collagen fibril distribution, cellular organization, and matrix composition that matches the specific tendon under investigation as success criteria for tissue engineered constructs. As there were no consistent molecular markers reported with tendon underloading, additional work is needed to identify specific molecular markers of aberrant cellular activity that could inform the development of engineered constructs that attempt to model underload-induced tendinopathy.

### Tendon overuse injuries in humans

Overuse-induced tendinopathy commonly occurs in professional athletes or in people whose professions require repetitive movements over long periods of time, and is most frequent in the Achilles, elbow, rotator cuff, and patellar tendons (Herod and Veres, 2018). In contrast to human tendon underloading studies, there are few studies that examine the effects of overuse on tendon morphology, ECM, or cell behavior which may be attributed to several issues including the fact that there is likely a “threshold” response that varies from person to person, which makes clinically defining “overuse” difficult and the fact overuse injuries can take a long time to develop, which makes linking activity to clinical outcomes challenging. Nonetheless, elite athletes provide the best evidence of the effects of overuse on tendon function. The patellar tendons of volleyball players diagnosed with patellar tendinopathy (i.e., “jumper’s knee”) exhibited increased CSA (19%), decreased stiffness (32%), and decreased elastic modulus (29%) compared to their non-dominant healthy patellar tendon (Helland et al., 2013). Pain is a common symptom of overuse tendinopathy (Swank et al., 2022; Kakade, 2023) and is associated with increased innervation within tendinopathic patellar tendon of athletes from various sports (Lian et al., 2006) suggesting that increased sensory nerve ingrowth may play role in symptomatic pain felt in overuse-induced tendinopathies; however the cause of this nerve ingrowth is unknown.

**FIGURE 3:**
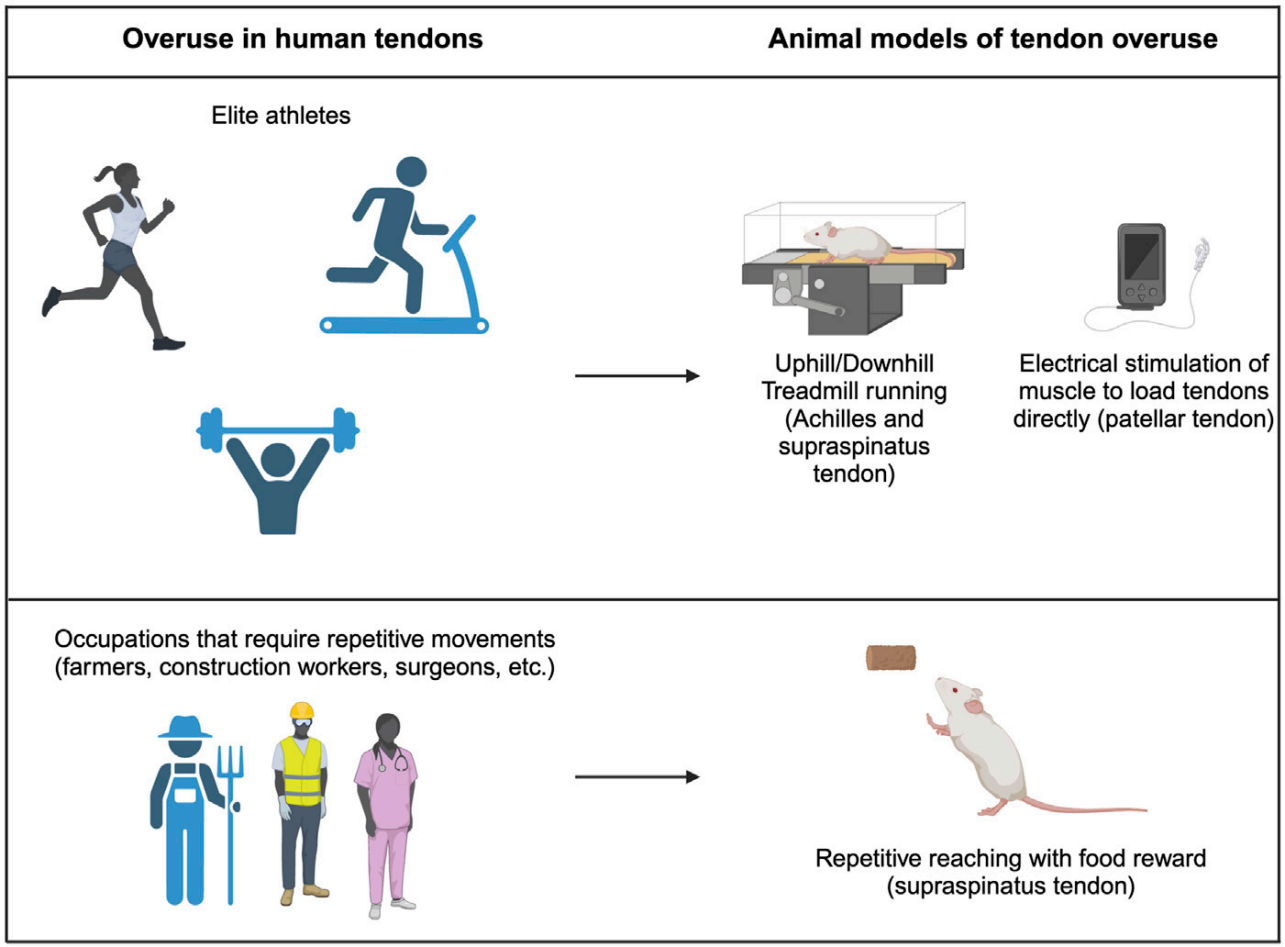
Tendon overuse in humans and corresponding animal models. Tendon overuse injuries in humans are best exemplified by elite athletes. Corresponding animals models use uphill and downhill treadmill running to cause overuse of the supraspinatus and achilles tendons. Additionally, direct electrical stimulation can be used to directly load patellar tendons. Physically demanding jobs that require repetitive movements such as farmers, construction workers and chefs frequently have report overuse tendon injuries which are best modeled by repetitive reach rodent models.

In addition to the challenges mentioned above, the lack of clinical presentation and samples gathered during early phases of overuse tendinopathy also hinders the field’s ability to understand the complex etiology and pathological progression in human patients. In the next section, we identify common outcomes from *in vivo* animal models of tendon overuse that mimic human activities (Figure 3). These models can give provide insight into the early pathological changes that occur with tendon overuse and highlight important considerations for tissue engineering applications. The animal models, specific tendons, and outcomes of all studies outlined below are provided in Table 2.

**TABLE 2:** Models of tendon overuse and their reported outcomes used to identify hallmarks of tendon overuse. Gray boxes indicate outcome was not reported.

Model	Animal	Tendon	Cellular density	Cellular morphology	Extracellular matrix organization	Collagen fibril size	Collagen fibril number	Tendon size	Inflammation markers	Chondrocyte markers	Mechanical properties	Material properties	Vasculature	References
Declined Treadmill	Rat	Achilles						No difference in width, thickness, or area			No difference in maximum load	No difference in maximum stress, modulus, or stress relaxation		Huang et al. (2004)
	Rat	Achilles	Increased	Rounded	Disorganized						Decreased stiffness and ultimate strength	No difference in stress relaxation		Ng et al. (2011)
	Rat	Achilles	No difference		Disorganized			No difference in CSA			No difference in maximum load	No difference in maximum stress	Increased	Kaux et al. (2013)
	Rat	Supraspinatus								Increased				Archambault et al. (2007)
	Rat	Supraspinatus	No difference	Rounded	Disorganized								No difference	Scott et al. (2007)
	Rat	Supraspinatus							Increased					Smozor et al. (2006)
	Rat	Supraspinatus	Increased	Rounded	Disorganized			Increased CSA				Decreased maximum stress and modulus		Soslowsky et al. (2000)
Inclined Treadmill	Horse	SDFT						No difference in CSA						Birch (2007)
	Horse	SDFT						Increased CSA						Kasashima et al. (2002)
	Rat	Achilles	Increased	Rounded	Disorganized			No difference in CSA	No difference					Glazebrook et al. (2008)
	Rat	Achilles	No difference		Organized			No difference in diameter				Increased maximum stress and modulus		Heinemeier et al. (2012)
	Rat	Achilles	No difference	No difference	No difference									Dirks et al. (2013)
	Rat	Achilles	Increased					Increased thickness	No difference				Increased	de Mello Malheiro et al. (2009)
	Rat	Achilles	Increased		Disorganized									Pingel et al. (2013)
	Rat	Achilles	Increased		Disorganized									Silva et al. (2011)
	Rat	Achilles	Increased	Rounded	Disorganized					Increased				Xu et al. (2016)
Treadmill (incline/decline not reported)	Rat	Achilles			Disorganized									Franchi et al. (2013)
Electrical Stimulation	Mouse	Achilles			No difference			No difference				No difference in maximum stress and modulus		Rezvani et al. (2021)
	Rabbit	Achilles	Increased	Rounded	Disorganized								Increased	Andersson et al. (2011)
	Rabbit	Achilles	No difference	No difference	No difference				No difference					Archambault et al. (2000)
	Rat	Achilles	Increased	Rounded	Disorganized				Increased					Cho et al. (2011)
	Rat	Achilles			No difference									Heinemeier et al. (2007)
Repetitive reaching	Rat	Flexor digitorum	Increased	Rounded					Increased		Decreased grip strength			Barbe et al. (2003)
	Rat	Flexor digitorum	Increased	Rounded	Disorganized				Increased		Decreased grip strength		Increased	Fedorczyk et al. (2010)
	Rat	Flexor digitorum	Increased	Rounded	No difference				Increased		Decreased grip strength		No difference	Kietrys et al. (2012)

### *In vivo* animal models of tendon overuse

#### Forced treadmill running

Forced treadmill running is frequently used to mimic overuse-induced tendinopathy seen in human runners, particularly of the Achilles tendon. In rodents, treadmill overuse models report no changes in the CSA of the Achilles tendon with uphill (Glazebrook et al., 2008; Kaux et al., 2013) or downhill running (Huang et al., 2004; Kaux et al., 2013), suggesting no large structural changes as a result of overuse. In rat Achilles tendons, 12 weeks of treadmill running on a 10° incline increased both the elastic modulus (41%) and failure stress (35%) with no changes in collagen fiber alignment or evidence of tearing (Heinemeier et al., 2012), suggesting that uphill running may actually provide positive adaptions rather than cause tissue damage. Indeed, 9 weeks of 15° inclined treadmill running resulted in no changes in cellular density or collagen fiber dispersion in rat Achilles tendons (Dirks et al., 2013). In contrast, other uphill rat running studies examining the Achilles tendon report evidence of tendinopathy indicated by hypercellularity and rounded nuclei (De Mello Malheiro et al., 2009; Silva et al., 2011; Pingel et al., 2013; Xu et al., 2016), increased proteoglycan content (Silva et al., 2011), disorganized collagen matrix (Franchi et al., 2013; Pingel et al., 2013; Xu et al., 2016), microtears (Silva et al., 2011) and elevated expression of chondrocyte markers including SRY-box transcription factor 9 (*Sox9*), aggrecan (*Acan*), and bone morphogenic protein-2 (*Bmp2*) at both the gene and protein level (Xu et al., 2016). Similar to uphill running, one study reported that downhill treadmill running for 1 h/day, 7 days/week for 8 weeks on a 20° decline decreased both stiffness (58%) and max load (53%) which, coupled with rounded nuclei and increased disorganization of the collagen matrix, indicating the development of overuse tendinopathy in rat Achilles tendon (Ng et al., 2011). However, another study employing downhill treadmill training (17 m/min, 10° decline, 1 h/day 5 days/week) reported no changes in mechanical properties or evidence of tendon degeneration in rat Achilles tendons after 16 weeks (Huang et al., 2004), supporting the notion that an ill-defined “threshold” of overuse may be required to trigger downhill induced tendinopathy. Interestingly, treadmill running in horses increased the superficial digital flexor tendon CSA (Kasashima et al., 2002), suggesting a species-specific effect of overuse on tendon hypertrophy. Downhill running employs more eccentric loading and poses greater risk to tendon injury than concentric exercises like uphill running (Friden and Lieber, 1992) and may serve as a better model of over-load induced tendinopathy, though this requires further investigation and refinement.

In addition to the Achilles tendon, many investigators have examined the effects of downhill treadmill running on the supraspinatus tendon. While this model does not recreate human supraspinatus tendon use or loading as humans do not bear weight on their shoulders while running, similar degenerative changes are seen in the supraspinatus tendon of animal after treadmill running as are observed in human cases of supraspinatus tendinopathy (Seitz et al., 2011). Eight weeks of treadmill running at a 10° decline induced a pathological response marked by increased cellularity and cell rounding, disrupted collagen organization, and decreased maximum stress (43%) and elastic modulus (49%) in the rat rotator cuff (Soslowsky et al., 2000), compared to cage activity controls. Other studies reported increased collagen fibril disorganization in Sprague-Dawley rats after 12 weeks (1 km/h, 11° decline, 1 h/day, up to 16 weeks) (Scott et al., 2007), elevated expression of the inflammatory marker gene nitric oxide synthase (Sprague-Dawley rats, 1 km/h, 10° decline, 1 h/day, 5 days/week, 4 weeks) (Szomor et al., 2006) and increased expression of the chondrocyte markers *Sox9* and *Acan* (Sprague-Dawley rats, 1 km/h, 10° decline, 1 h/day, 5 days/week, 4 weeks) (Archambault et al., 2007), which was interpreted as overload-induced fibrocartilage differentiation of tenocytes. Combined, these studies suggest that downhill treadmill running in rodents is sufficient to drive the development of overuse tendinopathy of the supraspinatus tendon.

#### Direct loading

Direct tendon loading by electric stimulation of attached muscles is a common method used to mechanically stimulate tendons which allows the operator fine control over the frequency and magnitude of the load (Archambault et al., 2000; Heinemeier et al., 2007; Andersson et al., 2011; Cho et al., 2011; Rezvani et al., 2021). Direct stimulation of muscle contraction resulted in increased disorganization of the collagen matrix and increased cellularity in the Achilles tendons of both rats and rabbits (Andersson et al., 2011; Cho et al., 2011). Interestingly, stimulation of isometric, eccentric, and concentric muscle contractions produce different gene expression responses in the rat Achilles tendon. Specifically, eccentric contractions increased mRNA expression of collagen-promoting growth factors including connective tissue growth factor (*Ctgf*), whereas eccentric and concentric contractions increased mRNA expression of type 3 collagen (*Col3a1*) indicating different types of muscle loading promote different patterns of gene expression (Heinemeier et al., 2007). However, no changes in collagen organization, CSA, maximum stress, or elastic modulus were seen when comparing eccentric vs. concentric contractions in a mouse Achilles tendon (Rezvani et al., 2021), which suggests that changes in gene expression may not result in large-scale structural changes to the tendon.

#### Repetitive reaching

To more accurately represent repetitive movement in occupational settings that can lead to overuse tendinopathies, a rat model using voluntary exertions in a repetitive forepaw and wrist-intensive task was developed in which the rats have to reach through a narrow tube to retrieve food pellets (Barr et al., 2000; Barr and Barbe, 2002). Both high repetition, high force and low repetition, negligible force repetitive reaching studies report increased expression of inflammatory markers (e.g., interleukin-Iβ and -1α, cyclooxygenase-2), increased numbers of resident macrophages, in rat flexor tendons compared to contralateral controls (Barr et al., 2000; Barbe et al., 2003; Fedorczyk et al., 2010). Combined, these studies point towards a substantial inflammatory component during repetitive overuse tendinopathy.

### Hallmarks of tendon overuse

*In vivo* animal models of tendon overuse consistently report increases in cell density, which may occur due to increased immune cell recruitment to the tendon given the elevated inflammatory cytokine expression reported in some studies and lack of evidence for tenocyte proliferation *in vivo* in response to increased inflammation; however, it is important to note that the specific cause of hypercellularity noted in these models is still unknown. As such, deciphering the composition of increased cellular density will be imperative to accurately design and evaluate engineered tendon constructs. Additionally, tenocytes in overloaded tendons are typically rounder and enlarged compared to healthy tenocytes, and coupled with elevated cartilage markers, this suggests the potential that tenocytes become more chondrocyte-like with overuse. In terms of ECM composition and integrity, collagen tearing and disruptions to the organized matrix are commonly reported among overused tendons. Increases in CSA are thought to be a hallmark of an overused tendon but treadmill running models have been unable to replicate this, and other models (direct loading, repetitive reaching, etc.) do not report changes in CSA even in the presence of other tendinopathic hallmarks. Interpreting changes in CSA of engineered constructs should therefore be done in concert with the evaluation of other tendinopathic markers (e.g., changes in cellularity, inflammatory markers, etc.) to delineate whether an increase in CSA is a result of hypertrophy (growth), inflammation (swelling), or fibrotic processes. Moreover, it is likely that the tendon response to load is dynamic over time such that alterations that appear pathological in the beginning may lead to long-term physiological adaptation. For example, it is well known that skeletal muscle adaptation to mechanical load involves an initial inflammatory response that is required to initiate muscle growth (Gomez-Cabrera et al., 2016). How this process is mediated in tendon is unknown, therefore longitudinal studies are needed to determine whether measured inflammatory responses in tissue engineered tendon constructs are indicative of adaptive remodeling or pathological progression.

While attempts to generate healthy tissue engineered replacement tissue should avoid the tendinopathic hallmarks described above, MPS aiming to model overuse tendinopathy should verify that the platform recapitulates pathological changes seen in *in vivo* studies including ECM disorganization, hypercellularity, tenocyte rounding, and impaired functional properties compared to healthy tendons. Tendon mimetics should also consider alterations in the expression of chondrogenic (Aggrecan, Sox9, etc.) or inflammatory (TNFα, IL-1a, IL-1b, etc.) markers that could signify a tendinopathic response. Similar to underloading, there is a clear difference in how different tendons respond to overuse. It is critical for tissue engineering efforts to take these tendon-specific responses into consideration and to design and evaluate constructs with specific tendons as the comparator.

## Discussion

In this review, we have described *in vivo* models of tendon underloading and overuse and identified hallmarks of tendon pathology that tissue engineering platforms can use to model against in the context of the development of engineered replacement tissues to replace damaged tissue or augment tendon healing, or to incorporate in the context of MPS that seek to mimic specific types of degenerative tendinopathy. As described above, tendinopathy can result from both under and overuse, and while both alterations in mechanical strain led to similar outcomes, the specific mechanisms and hallmarks of each type of tendon degeneration are different. For example, overuse tendon injuries are frequently associated with inflammation, whereas the current models of underloading do not point towards a role for inflammation in underload-induced degeneration. This is a crucial point to consider for both attempts to engineer replacement tissues and for the design of MPS models: in the case of tissue engineered tendon, distinct markers of underuse (smaller collagen fibril area and diameter) or overuse (chondrocyte and inflammatory markers) can be used to fine-tune mechanical stimulation to promote healthy cellular behavior vs. damage whereas MPS should attempt to model specific types of tendinopathy (overuse or underload) in order to have more definable design benchmarks and interpretable outcome measures.

Notably absent from both the current review and the literature at large is a thorough understanding of the cellular and molecular mechanisms that maintain tendon homeostasis in response to physiological load. It is well established that appropriate loading is required to maintain homeostasis (Bohm et al., 2015), and as we have highlighted throughout this review, too much or too little mechanical stimulation can lead to degeneration, making it crucial to identify the “correct” amount of loading to maintain the health of engineered constructs. However, the precise cellular mechanisms that allow tenocytes to translate physiological load into a tissue wide adaptation response is unknown. This lack of information makes evaluating the effectiveness of various loading regimes in the context of tissue engineering extremely difficult, as defining the fine line between “good” load and “bad” load is currently not possible. This is further complicated by the fact that there are clearly tendon-specific and species-specific responses to loading. As such, there is no “universal” threshold for proper mechanical stimulation needed to maintain tendon homeostasis, or that can be prescribed to accurately mimic *in vivo* tendon behavior in tissue engineered constructs. In the absence of such information, tissue engineering attempts to generate new tendon tissue should aim to recapitulate the native ECM composition, cellular composition and organization, and mechanical properties of *specific tendons* by using the native tendon as the “gold standard” comparison to evaluate success. For example, tissue engineering studies frequently report an increase in the expression of tenogenic markers within a given system as a “good” outcome; however, these comparisons are typically made in reference to either earlier timepoints in the study or to biomaterials of different composition and not to a specific tendon. As increased expression of tendon markers, including the tenogenic transcription factor scleraxis, can be homeostatic or pathological depending on the context (Kendal et al., 2020; Morita et al., 2021), it is critical for tissue engineers to consider the context and to strive to recreate gene and/or protein expression patterns that are rooted in native tendon tissue rather than changes within an engineered system. Similarly, the effects of loading and tendinopathic progression must be considered in a tendon-specific manner for tissue engineering or MPS efforts that aim to model tendon degeneration. Rather than attempting to broadly mimic tendon degeneration, investigators should focus on a specific tendon (e.g., Achilles or supraspinatus) and type of load-induced pathology (e.g., underload or overuse) in order to have specific, definable points of comparison.

An additional aspect to consider for tissue engineering efforts to generate whole tendon tissues is that there is enormous spatial variation within a tendon at both the cellular and structural scale. For example, the distal portion of the Achilles tendon of long distance runners is significantly larger compared to non-runner controls (Magnusson and Kjaer, 2003), which suggests distinct loading requirements and subsequent responses to loading. Indeed, the distal, midsubstance, and proximal portions of the mouse Achilles tendon display region-specific transcriptomic signatures and mechanical properties, emphasizing the need to consider spatial specific adaptation responses to loading (Disser et al., 2022). Recent single-cell RNA sequencing of tendon cells has also revealed vast heterogeneity among tenocytes, identifying multiple subpopulations during homeostasis and healing both within a given tendon and between different types of tendons (De Micheli et al., 2020; Kendal et al., 2020; Ackerman et al., 2022; Nichols et al., 2023; Mimpen et al., 2024b). Considering that different anatomical tendons experience different types of loads (tensile, compressive, and shear stresses), combined with both spatial and cellular heterogeneity within the tendon, tissue engineering efforts that aim to mimic overuse or underuse tendinopathy should consider how this mechanical, cellular, and matrix heterogeneity may influence the onset of degenerative changes.

### Limitations of *in vivo* animal models of tendon loading

Not all animal models of tendon loading were able to consistently recapitulate the same degenerative changes seen in human cases, some of which can be attributed to inherent limitations of animal models. Many animal models (e.g., forced treadmill running and hindlimb suspension) cause the animals to perform unnatural or difficult tasks, which leads to a systematic stress response that likely influences the tendon mechanoresponse. Degenerative changes seen in these models could therefore be the result of elevated systemic inflammation in concert with loading, rather than solely load-induced.

While animal models provide an attractive framework to manipulate loading induced tendinopathies in a controlled and reproducible manner, many lab animals are quadrupeds and therefore do not experience the same magnitude or type of load as humans (Hast et al., 2014). Furthermore, there are known molecular differences between animals and humans. For instance, rodents do not possess a homologue of the *MMP1* human gene (Foley and Kuliopulos, 2014), which complicates our understanding of how *MMP1* contributes to ECM remodeling in *in vivo* models of loading. There are also important differences in the anatomy and mechanical properties of animal tendons compared to human tendons, which are discussed in-depth in a recent review (Burgio et al., 2022). For example, the rat possesses the most similar anatomical structure of the rotator cuff to humans and therefore may serve as a better point of comparison than other rodent models. Another important difference is in the hierarchical structure of tendons. While the tendons of small mammals like mice are relatively simple, consisting of a collagen matrix surrounded by the epitenon, larger mammals have a more complex hierarchical structure that includes the IFM. If and how these other compartments of the tendon may be involved in the response to tendon loading therefore cannot be ascertained using mouse models.

Furthermore, many *in vivo* animal studies of tendon loading report limited outcome measures, typically focusing on changes in mechanical properties or largely superficial characterizations of cellular, molecular, and ECM changes. As discussed above, this makes interpretation of results difficult, as there is no one singular hallmark of tendon health or degeneration. For example, unloading via Botox increased the elastic modulus of rat Achilles tendons (Khayyeri et al., 2017) which authors interpreted as a degenerative response due to an association with disorganized collagen matrix. In contrast, increased modulus of the Achilles tendon from inclined treadmill trained rats was considered nonpathological; however, no other metrics were reported so this interpretation cannot be fully evaluated (Heinemeier et al., 2012). In order to clarify outcomes and understand the effects of load on tendon health from *in vivo* animal models as well as in tissue engineering efforts, multiple outcomes must be examined.

### Perspectives and future directions

While tissue engineering approaches hold great promise to improve tendon health and healing, there is still substantial work to do to best leverage these techniques and technologies. The vast heterogeneity and disparate outcomes noted in the animal models of tendon loading highlighted in this review speak to an urgent need to better understand specific types of load-induced tendinopathy in anatomically-distinct tendons, as well as the cellular and molecular mechanisms responsible for driving different degenerative tendon pathologies. The past few years have seen an enormous effort dedicated to characterizing the cellular heterogeneity within different tendons using both genetic reporter mice and new technologies including single-cell RNA-sequencing and spatial transcriptomics, in both healthy and diseased tendons across multiple species (Garcia-Melchor et al., 2021; Ackerman et al., 2022; Korcari et al., 2023; Nichols et al., 2023). Tissue engineers can leverage these transcriptomic markers and signatures to identify cell populations that should be present in engineered constructs or mimetics and to ensure that engineered systems promote the desired cellular behavior. Additionally, while most tissue engineering efforts use relatively simple constructs to represent tendon, most frequently with cells embedded in simple hydrogels or biomaterials, tendon is not a simple or homogenous tissue. As mentioned above, there is great spatial heterogeneity not only in the types of cells present but also in the ECM and mechanical loads experienced along the length of a given tendon. Recently, several investigators have characterized these properties in native tendon, which can be directly translated into tissue engineered constructs. For example, a study characterized the transcriptomic and mechanical properties along the length of the rat Achilles tendon, and gave defined CSA, stress, and elastic moduli for three defined regions (proximal, middle, and distal) (Disser et al., 2022). While more work is needed to perform similar characterization in other tendons, and across species, tissue engineering efforts can utilize these metrics to ensure engineered constructs more faithfully recapitulate the properties of native tendons.

Finally, while most tissue engineering efforts to date have focused on generating the collagen matrix of tendons, few have attempted to model the hierarchical structure of native tendons. The components and properties of other compartments within the tendon have been poorly defined historically which has precluded their inclusion in tissue engineered constructs. Recent work to define both the IFM (Thorpe et al., 2016) and the epitenon (Nichols et al., 2023) at both the cellular and molecular level has provided a wealth of markers and metrics that tissue engineers could incorporate into engineered tendon constructs. Because it is difficult to study or image these structures *in vivo,* it is still unknown how different tendon compartments interact, which represents an excellent opportunity for tissue engineers to create systems in which these relationships can be interrogated. Generation of more complex engineered constructs will not only enhance our understanding of fundamental tendon biology but will also increase the physiological relevance and clinical translatability of tissue engineered tendon.

## Conclusion

Despite the significant healthcare burden tendon injuries pose, there are no clinically approved tissue engineering constructs to replace or regenerate degenerative tendons. Current approaches have largely failed to mimic native tendons, in part due to the lack of benchmarks to evaluate the effects of mechanical loading. In this review, we have defined cellular, molecular, structural, and mechanical hallmarks of tendon underloading and overuse from various *in vivo* animal models of loading as a guide to help inform tissue engineering efforts. Moreover, we urge tissue engineers to consider the tendon-specific requirements for mechanical loading, as well as the cellular, mechanical, and structural complexity of individual tendons. Focusing on the precise cellular and molecular mechanisms in which tendons translate mechanical stimulation to a physiological, rather than pathological, tissue-wide adaptation response will ultimately improve the design of engineered constructs and hopefully help expedite clinical translation.
